# Distractibility during retrieval of long-term memory: domain-general interference, neural networks and increased susceptibility in normal aging

**DOI:** 10.3389/fpsyg.2014.00280

**Published:** 2014-04-07

**Authors:** Peter E. Wais, Adam Gazzaley

**Affiliations:** Departments of Neurology, Physiology and Psychiatry, Center for Integrative Neurosciences, University of California, San FranciscoSan Francisco, CA, USA

**Keywords:** distraction, long-term memory, categorization, top-down control, episodic memory

## Abstract

The mere presence of irrelevant external stimuli results in interference with the fidelity of details retrieved from long-term memory (LTM). Recent studies suggest that distractibility during LTM retrieval occurs when the focus of resource-limited, top-down mechanisms that guide the selection of relevant mnemonic details is disrupted by representations of external distractors. We review findings from four studies that reveal distractibility during episodic retrieval. The approach cued participants to recall previously studied visual details when their eyes were closed, or were open and irrelevant visual information was present. The results showed a negative impact of the distractors on the fidelity of details retrieved from LTM. An fMRI experiment using the same paradigm replicated the behavioral results and found that diminished episodic memory was associated with the disruption of functional connectivity in whole-brain networks. Specifically, network connectivity supported recollection of details based on visual imagery when eyes were closed, but connectivity declined in the presence of visual distractors. Another experiment using auditory distractors found equivalent effects for auditory and visual distraction during cued recall, suggesting that the negative impact of distractibility is a domain-general phenomenon in LTM. Comparisons between older and younger adults revealed an aging-related increase in the negative impact of distractibility on retrieval of LTM. Finally, a new study that compared categorization abilities between younger and older adults suggests a cause underlying age-related decline of visual details in LTM. The sum of our findings suggests that cognitive control resources, although limited, have the capability to resolve interference from distractors during tasks of moderate effort, but these resources are overwhelmed when additional processes associated with episodic retrieval, or categorization of complex prototypes, are required.

## Introduction

A growing body of research shows that the presence of irrelevant information, which is a common factor in our real-world environment, diminishes performance in visual working memory (WM) (Rainer et al., 1998; Lavie, 2005; Zanto and Gazzaley, 2009; Clapp et al., 2010) and in the retrieval of details from long-term memory (LTM) (Wais et al., 2010, 2012a; Wais and Gazzaley, 2011). The ability to remain focused on relevant visual stimuli in the presence of distractors is thought to depend on selective visual attention (Desimone, 1998; Lavie and de Fockert, 2005). Neuroimaging evidence suggests that perceptual processing of visual distraction interferes with connectivity of functional networks that guide visual attention to achieve memory goals. Moreover, the effect of visual distraction on performance increases with normal aging in the domains of WM (Gazzaley et al., 2005a; Berry et al., 2009) and LTM (Wais et al., 2012b).

We review here the implications of recent findings from behavioral and neuroimaging results that the presence of visual distraction negatively impacts the fidelity of LTM retrieval. Additionally, we discuss results that suggest the negative impact of distractibility on details retrieved from LTM is a domain-general phenomenon—a finding that suggests a direct relationship between the increased susceptibility to visual distraction in normal aging and impairment in categorization abilities.

### Distraction reduces fidelity of long-term memory retrieval

Previous behavioral studies have shown that engagement in a secondary cognitive task during LTM retrieval (i.e., divided attention) interferes with free recall (Fernandes and Moscovitch, 2000) and source memory (Troyer et al., 1999). Our motivation was to investigate the impact of distraction by entirely irrelevant visual information on a participant's singular goal of retrieving episodic details from LTM. Because attentional resources are limited (Pashler and Shiu, 1999), the top-down effort required to retrieve details relevant for memory goals may suffer when incidental attention to the irrelevant visual information diverts resources away from LTM goals. Although this diversion would be clearly driven by bottom-up processes, because there are no top-down goals to attend to the visual stimuli, excessive demands on brain regions and networks in common across these processes may result in substantially diminished fidelity of LTM.

Our experimental approach used in several studies was to cue participants to recall previously studied objects during blocks when their eyes were closed, or were open and irrelevant visual information was present. We hypothesized that because visual imagery in support of episodic retrieval utilizes the same limited-capacity lateral occipital cortex (LOC) buffers that are involved in processing external visual stimuli (De Fockert et al., 2001; Lavie, 2005), as well as overlapping cognitive control networks (Blumenfeld and Ranganath, 2006), visual stimulation during a retrieval effort would disrupt the access to or fidelity of details about a prior experience stored in LTM. This may be the driving force behind common acts of looking away or closing one's eyes when engaged in effortful recollection (Glenberg et al., 1998), reflexive efforts that may serve to block interference between irrelevant external information and recalling details from memory.

### Results for visual distraction

**Figure 1:**
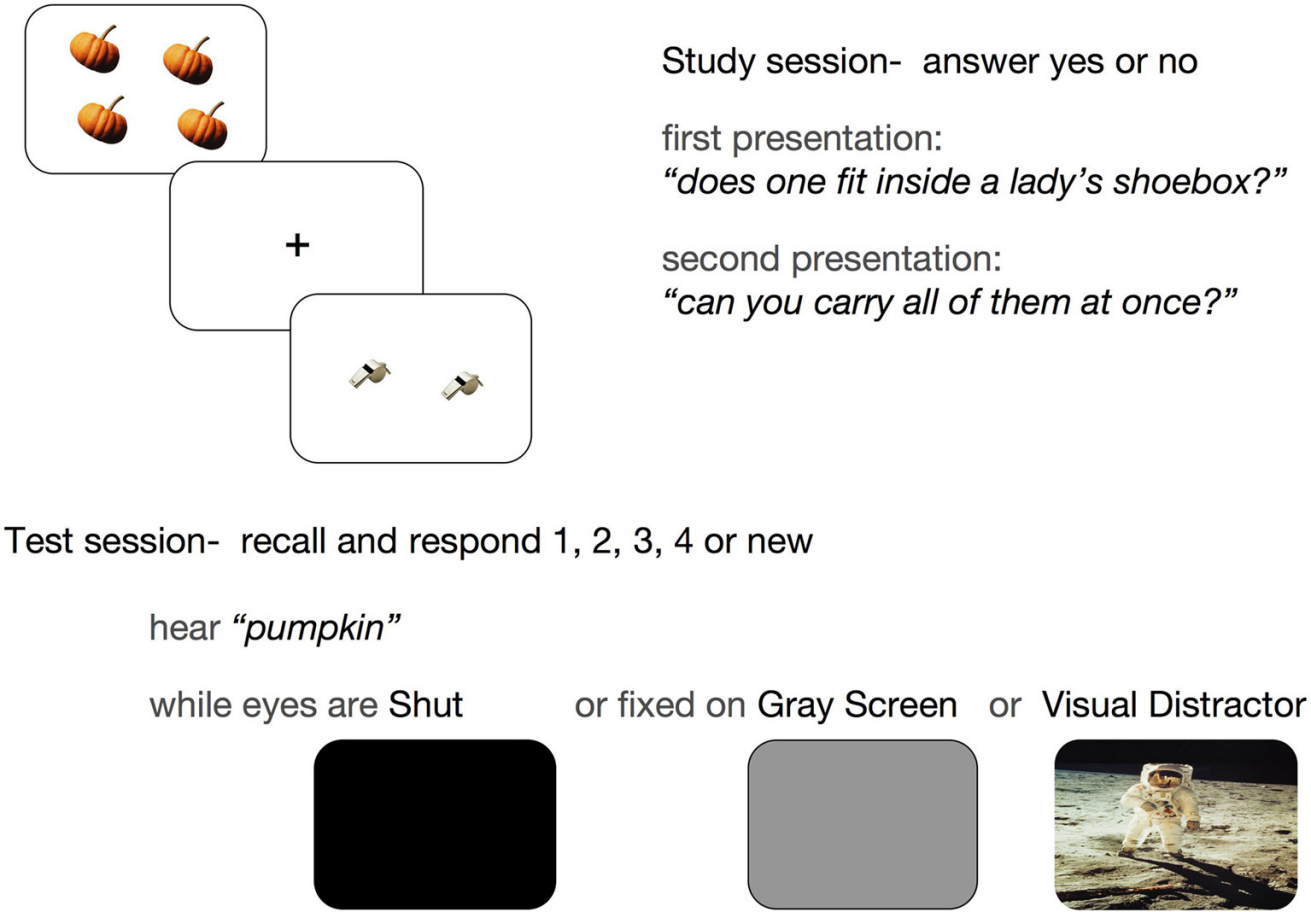
**Experimental paradigm**. A schematic of the procedure shows the study session, when participants answered two incidental questions about each of 168 images (3 s per presentation), and the test session, when auditory cues described 168 targets and 36 lures in singular form (2.5 s per presentation, 10.0 s inter-trial interval). Participants' recall was tested during in three conditions: SHUT, GRAY, and Visual Distractor (VD) (Wais et al., 2010).

In a behavioral study, participants studied images of common objects during two incidental encoding tasks, and, after a 1-h retention interval, responded old or new to auditory cues for target and lure objects (Wais et al., 2010). During encoding, each object image displayed one to four copies of a common object from a three-dimensional perspective. During test blocks, an auditory cue described an object encoded in the previous session, or a novel (i.e., lure) object, in singular form. Participants were instructed to recall the count for the object described by the cue and give their answer by responding 1, 2, 3, 4, or “new.” Correct responses for the object count indicated retrieval of goal-relevant episodic information. Test blocks presented auditory cues for targets in three different conditions: when visual stimulation was nil (eyes closed: SHUT), when bottom-up processing was minimal (looking at a gray screen: GRAY), and when neutral, visual environmental stimuli were presented (Visual Distraction, or VD) (Figure 1). The visual stimuli appeared simultaneously with the presentation of the auditory cues, and participants were instructed to fix their gaze at the center of the computer screen during stimulus presentation in GRAY and VD trials.

**Figure 2:**
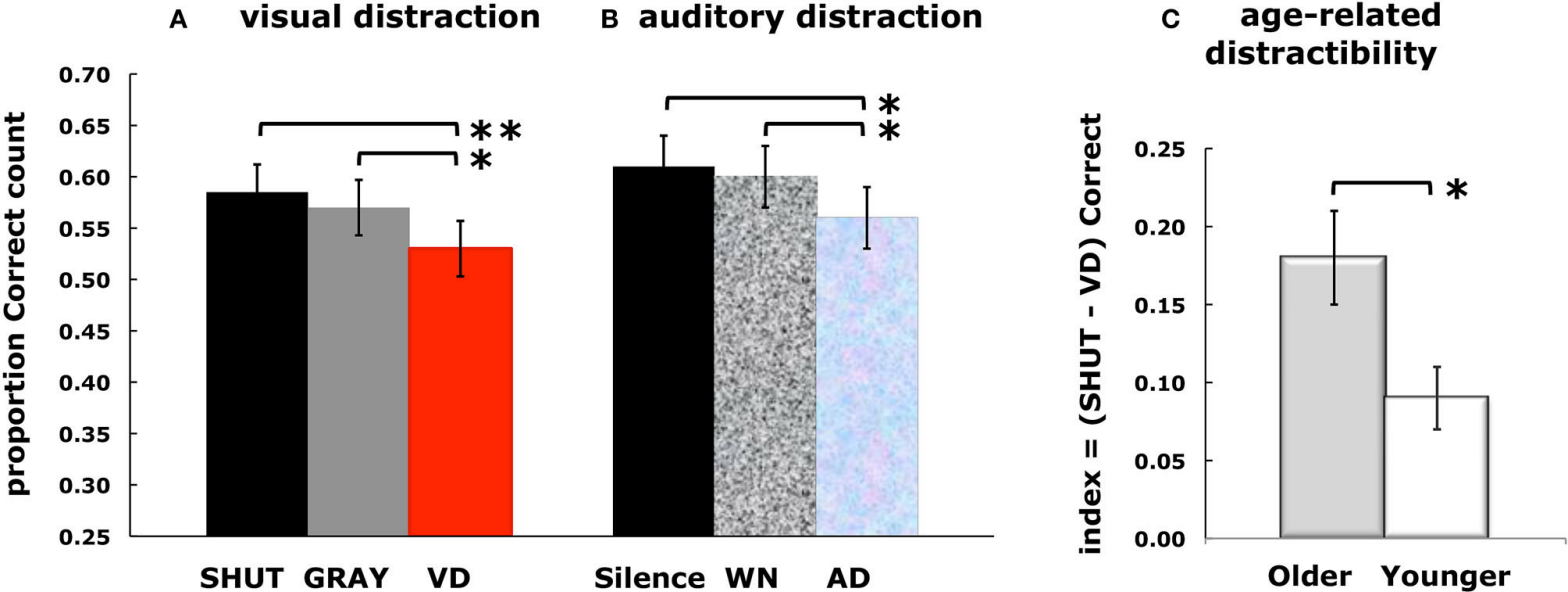
**Results from behavioral experiments**. Episodic retrieval scores are presented for three studies that used separate groups of participants. Each panel shows the negative influence of external distraction. For visual distractors **(A)**, the mean proportion of targets given responses with the correct count by a group of younger adults is diminished in the visual distraction (VD) condition relative to the SHUT and GRAY conditions. For auditory distractors **(B)**, the results from younger adults show the same pattern of diminished performance in the auditory distraction (AD) condition, relative to the silence and white noise (WN) conditions, as observed for visual distraction. A comparison between groups of younger and older adults **(C)** who completed the visual distraction paradigm revealed that the disruptive influence of distraction diminished the fidelity of episodic retrieval to a greater degree for older adults than younger adults. Error bars represent the standard error of the mean, ^*^p < 0.05, and ^**^p < 0.001.

Overall memory performance for each of the test conditions was indexed using an estimation of d' for each participant (mean overall *d*' = 2.10 ± 0.09), a measure that contrasts the hit rate for targets with the false alarm rate for lures (Macmillan and Creelman, 2005). A comparison across test conditions showed a main effect of condition, such that d' was greater for SHUT than both GRAY and VD (Table 1; Visual Distractors, younger adults). Comparison across conditions of the responses for the targets revealed a main effect of condition for the proportion given the correct count, and pair-wise tests showed that episodic retrieval during VD was significantly reduced compared to both SHUT and GRAY (Figure 2A).

**Table 1:** **Behavioral results for groups of younger and older adults**.

	**Visual distractors**			**Auditory distractors**		
	**SHUT**	**GRAY**	**VD**	**Silence**	**WN**	**AD**
**YOUNGER ADULTS**						
Proportion correct	0.59 (0.03)	0.57 (0.03)	0.53 (0.02)	0.61 (0.03)	0.60 (0.02)	0.56 (0.03)
Recognition d'	2.46 (0.13)	2.11 (0.14)	1.97 (0.10)	2.07 (0.12)	2.25 (0.10)	2.23 (0.12)
**OLDER ADULTS**						
Proportion correct	0.48 (0.02)	0.46 (0.03)	0.40 (0.02)			
Recognition d'	1.56 (0.14)	1.63 (0.13)	1.70 (0.12)			

Summaries for each of three experiments show group means for the proportion of targets given correct responses and for recognition d' (i.e., comparison of hit rate to false alarm rate) in each condition (standard error of the mean). Different groups of younger adults participated in experiments with either visual or auditory distractors: SHUT and Silence presented no external information during memory test trials; GRAY and white noise (WN) presented control stimuli; and visual distractors (VD) and auditory distractors (AD) presented external information irrelevant for the goal of episodic retrieval. A group of older adults completed a standardized neuropsychological battery, and scored within 2.0 standard deviations of their age-matched normative value, before participating in a visual distraction experiment using the same paradigm as the younger adults.

The results revealed that irrelevant visual stimuli presented during a memory test diminished the fidelity of details retrieved from LTM. This finding suggests that there is a critical role for cognitive control processes in minimizing the disruptive influence of irrelevant external information during episodic retrieval. Notably, the failure to inhibit the processing of distractions had also been shown in previous research to diminish accuracy in perception and visual WM (Lavie et al., 2004; Gazzaley et al., 2005b, 2008; Zanto and Gazzaley, 2009; Clapp et al., 2010).

Recent studies have distinguished between the impact of interference from distraction (entirely irrelevant information) and interruption (relevant information for a secondary task) on WM, and revealed that distinct neural mechanisms underlie these two types of interference (Clapp et al., 2010), as well as the presence of differential effects in aging (Clapp and Gazzaley, 2012). Our first study specifically explored the influence of distraction-related interference on LTM retrieval, as the visual stimuli in the VD condition were entirely irrelevant (i.e., participants were explicitly instructed to direct their undivided attention to the goal of responding to the memory test). Our findings of a decrement in episodic retrieval in the setting of distraction parallel the documented impact by interruption (dual-tasking) on LTM (Jacoby, 1991; Troyer et al., 1999; Fernandes and Moscovitch, 2000; Fernandes et al., 2006), but given the data from WM experiments, it is reasonable to hypothesize that distraction and interruption effects on LTM likely involve distinct neural mechanisms.

Our results also raise the possibility that two, non-mutually exclusive, neural mechanisms may underlie the impact of distraction on episodic retrieval. First, bottom-up, visual processing of external information may result in a decrease in the fidelity of internal representations of memoranda generated via visual imagery during the retrieval period, because both types of representations rely on overlapping regions of visual cortices. For example, the fidelity of details retrieved in imagery in response to memory task goals (i.e., the precise count of pumpkins on the studied image) could be diminished due to interference from processing concurrent, although irrelevant, visual information. In this example, recollection of details would be disrupted, yet a general assessment of recognition accuracy (i.e., are pumpkins old or new?) would not reveal an impact of distraction. Second, because attentional resources are limited (Pashler and Shiu, 1999), top-down effort required to retrieve memories when cued may suffer when incidental attention to the irrelevant visual information diverts resources away from LTM goals, resulting in diminished fidelity of episodic details. Interestingly, studies that examined effects of distraction in circumstances like eyewitness testimony have reported findings convergent with our results from trial-wise tests of cued recall. The findings showed recall for visual details was superior in eyes closed, relative to eyes open, conditions (Perfect et al., 2011; Vredeveldt et al., 2011) and support the interpretation that eye closure removes the cognitive load associated with monitoring the external environment.

### Results for auditory distraction

Another behavioral study utilized an experimental paradigm that paralleled the previous study, but substituted auditory distractors in place of visual distractors (Wais and Gazzaley, 2011). Because bottom-up processing of auditory stimuli and internal visual representations of items in memory are thought to be supported by discrete sensory cortices, our rationale for this next study was that if auditory distraction effects were present, then the convergence of these results with those from the prior study would suggest that external interference effects on episodic retrieval occur in a domain general manner. The experimental paradigm utilized written cues to probe recall of visual details of previously studied objects when participants were: (1) in complete silence, (2) exposed to white noise, or (3) exposed to ambient sounds recorded at a busy café. The target stimuli and encoding procedure in the current study with auditory distraction were identical to those in the previous study with visual distraction.

We examined the impact of auditory distraction on retrieval of visual memories, and then compared those results to our findings that revealed the impact of visual distraction on LTM retrieval. Estimates of d' were used in a comparison of overall performance (mean overall *d*' = 2.14 ± 0.08, Table 1; Auditory Distractors, younger adults), and there was no effect between the control and distraction conditions (i.e., Silence, White Noise, and AD). Comparison of the responses for the targets across conditions revealed a main effect of condition for the proportion given the correct count, such that episodic retrieval was significantly disrupted by auditory distraction (Figure 2B). Pair-wise comparisons showed that episodic retrieval during AD was reduced compared to both Silence and White Noise, with no significant difference between Silence and White Noise.

A direct comparison was performed between results of the auditory distractor study with the visual distractor study (Wais et al., 2010). In the auditory distraction experiment, the conditions for no distractor (Silence), control distractor (White Noise), and distractor (AD) were analogous, respectively, to eyes shut (Shut), eyes open with gray screen (Gray), and eyes open with complex natural scenes (VD) in the visual distraction experiment. Conditional correct scores computed the proportion of responses for a correct count given that an item was not forgotten [i.e., *p*(Correct)/(1-*p*(Forgotten)] and were used to compare performance with a mixed-design, 2 distractor modality (auditory, visual) × 3 condition (no distractor, control distractor, distractor) ANOVA. The results showed a main effect of condition such that retrieval of relevant visual details during the distractor conditions declined relative to both the no distractor and control distractor conditions. There was no difference in the pair-wise comparison between the no distractor and control distractor conditions. Critically, there was no main effect of distractor modality and no interaction between condition and distractor modality.

The comparison across the experiments revealed that there was no difference in effect between distractor modality: i.e., auditory and visual information irrelevant to the LTM goal induced equivalent interference effects on retrieval of task-relevant, visual details. In our results, the influence of distraction on episodic retrieval of visual details is, therefore, independent of the sensory domain of the distractor. Other studies that examined effects of visual and auditory distraction during eyewitness-like recall have found evidence for modality-specific interference (Vredeveldt et al., 2011) and particular susceptibility for visual distraction (Perfect et al., 2011). Compared to these findings, the domain generality of distractors' disruptive influence in our results may have to do with the high level of attentional demands in our trial-wise, time-constrained tests for retrieval of specific visual details. The disruptive impact on domain general processes could be explained by either top-down or bottom-up interference (which are not mutually exclusive). Specifically, LOC regions supporting visual imagery for the target images might be impacted by bottom-up influences from the multisensory processing of visual or auditory stimuli (Ghazanfar and Schroeder, 2006), or regions of the prefrontal cortex (PFC) that mediate top-down signals to visual pathway regions might be disrupted in a domain independent manner (Ranganath et al., 2004). Because there is no direct overlap in primary sensory regions, it is more likely that the former explanation is the cause of the distraction effect.

### Impact of visual distraction on episodic retrieval in older adults

Cognitive aging takes a toll on both the encoding (Ferguson et al., 1992) and retrieval (Hashtroudi et al., 1990) of information that forms our awareness of prior experiences—memories. Research aimed at characterizing the specific nature of LTM impairment has highlighted age-related deficits in retrieval of episodic information (Li et al., 2004) and suggests that older adults do not retrieve vivid, detailed information about prior episodes as effectively as younger adults (Craik, 2002). To explore the impact of visual distraction on LTM in older adults, we utilized the same experimental paradigm used previously with younger adults (Wais et al., 2010; Wais and Gazzaley, 2011).

In our study of older adults (Wais et al., 2012b), the incidental encoding procedure was the same for all target images (i.e., the study session), which held the detail and quantity of information equivalent for all test stimuli. Therefore, any impairment that existed in the older adults' ability to encode the details of studied stimuli (Chalfonte and Johnson, 1996) would impact each test condition equally (i.e., SHUT, GRAY, and VD). Furthermore, because the incidental encoding procedure and retention interval were the same as used previously with younger adults, the analysis could distinguish between a generalized age-related decline in LTM performance and a differential impact of visual distraction on episodic retrieval in older adults.

Overall recognition performance (mean overall *d*' = 1.63 ± 0.12) was compared between conditions using estimates of d' (Table 1; Visual Distractors, older adults). A mixed-design ANOVA (younger/older × SHUT/GRAY/VD) for estimates of d' revealed a main effect of age, no effect of condition, and an interaction of age and condition. The interaction of age and condition on d' reflected better performance by younger adults when visual distractors were not present: SHUT, young > old; GRAY, younger > older; and no difference between younger and older in VD.

A mixed-design ANOVA (younger/older × SHUT/GRAY/VD) compared conditional correct scores [i.e., for targets, *p*(Correct)/(1-*p*(Forgotten)] and revealed a main effects of age and of condition, as well as an interaction of age and condition. To interrogate this interaction, both within-group and between-group tests were performed. Pair-wise comparisons within the older adult group showed that retrieval of relevant visual details declined significantly in VD relative to SHUT and GRAY, and there was no difference between conditional correct scores for SHUT and GRAY. Between-group comparisons, which directly compared conditions for older and younger adults, revealed an aging-related decline in episodic retrieval in VD, while there were only trends for aging-related declines in SHUT and GRAY. This finding that older adults exhibited diminished detailed LTM in the setting of visual distraction is in contrast to the absence of an age-related change on overall recognition as the impact of distraction, thus establishing the selectivity of distractibility on episodic retrieval.

Further analyses used a distraction index to account for overall differences between age groups in the fidelity of LTM retrieval induced by distraction. For each older and younger participant, a distraction index was calculated for conditional correct scores (i.e., SHUT correct—VD correct). A greater index corresponds to greater disruption by distraction during episodic retrieval, that is to say greater distractibility. An independent samples test of the distraction index, assuming unequal variances, revealed greater distractibility in the older adults than the younger adults (Figure 2C). The result of the comparison of distractibility indices provides strong evidence that visual distraction disrupted retrieval of relevant details from LTM to a greater degree in older than younger adults.

Our interpretation of the results that show episodic retrieval in older adults is more susceptible to disruption by irrelevant, external information is that decline in performance was caused by interference on control processes mediating the selection of specific mnemonic details. Several explanations have been proposed for the selective decline in recollection in normal aging, including deficits in retrieving multiple features (Chalfonte and Johnson, 1996), in the vividness and complexity of visual imagery for prior experiences (Henkel et al., 1998), and in the ability to merge associations that form episodes (Naveh-Benjamin et al., 2003). These deficits all reflect diminished accessibility to specific details about prior experiences. A common feature influencing all of these deficits, including the results from the current study, may be an impact of interference on selection processes that support retrieval of detailed memories.

The current findings may reflect a more fragile top-down control network in older adults, even when the older participant's eyes were shut, which explains the trend of weaker episodic memory performance in SHUT compared to younger adults. Top-down control guiding the selection of relevant details during episodic retrieval would then be further compromised by interference from visual distraction, resulting in a larger cumulative impact on memory retrieval processes in older adults when irrelevant, external information was present. Further research using neuroimaging will be required to elucidate the impacted neural networks that generate increased susceptibility to interference in the presence of visual distraction, which in turn underlies the weakened fidelity of LTM in normal aging.

### Neural mechanisms underlying distractibility during LTM retrieval

In an fMRI experiment (Wais et al., 2010), we examined the neural networks that support episodic retrieval involving visual imagery and how functional connectivity in those networks is impacted by the presence of irrelevant visual information. The study used the same paradigm and stimuli from the related behavioral experiment and included minor adjustments in stimulus timing to accommodate the fMRI procedure. Evaluation of the neural basis of interference effects using fMRI involved first contrasting univariate data in the SHUT condition associated with trials when the correct count was given (i.e., episodic retrieval) vs. trials when an incorrect count was given. This contrast enabled the identification of brain regions of interest associated with successful episodic retrieval, which were then used as seeds in a functional connectivity analysis to characterize neural networks that supported episodic retrieval in the absence of external distraction. Subsequent contrasts between the SHUT and VD conditions explored the neural basis of interference induced by the presence of irrelevant visual information. We hypothesized that retrieval of the details of the studied images would be impaired when visual distraction was present during the memory test, and that this interference would be mediated via disruption of functional neural networks involving memory regions in the medial temporal lobe (MTL), control regions in the PFC and stimulus-selective regions in the lateral occitpital cortex (LOC).

The performance results for the participants tested in the MRI scanner replicated the previous behavioral study: the fidelity of details retrieved from LTM was diminished in the presence of visual distraction, relative to the eyes shut condition. The first step in the fMRI analysis was to identify regions in a whole-brain contrast where activity increased in association with correct, relative to incorrect, cued recall responses in the condition that was free of influence from external visual stimuli (i.e., SHUT). Three regions revealed increased activity in support of episodic retrieval during SHUT: the left hippocampus, the right hippocampus, and the left LOC (all *p-corrected* < 0.05). Of note, the left LOC region that supported episodic retrieval in SHUT overlapped with the object-selective ROI identified in a separate object localizer block. Activity increased in this LOC region above the fixation baseline despite eyes being closed, and no increases were observed in other LOC regions in association with either SHUT correct or SHUT incorrect responses, relative to the forgotten items or baseline fixation. This pattern of increased activity in a stimulus-selective area of the left LOC could not have been associated with processing external visual stimuli because the participants' eyes were closed during these trials.

**Figure 3:**
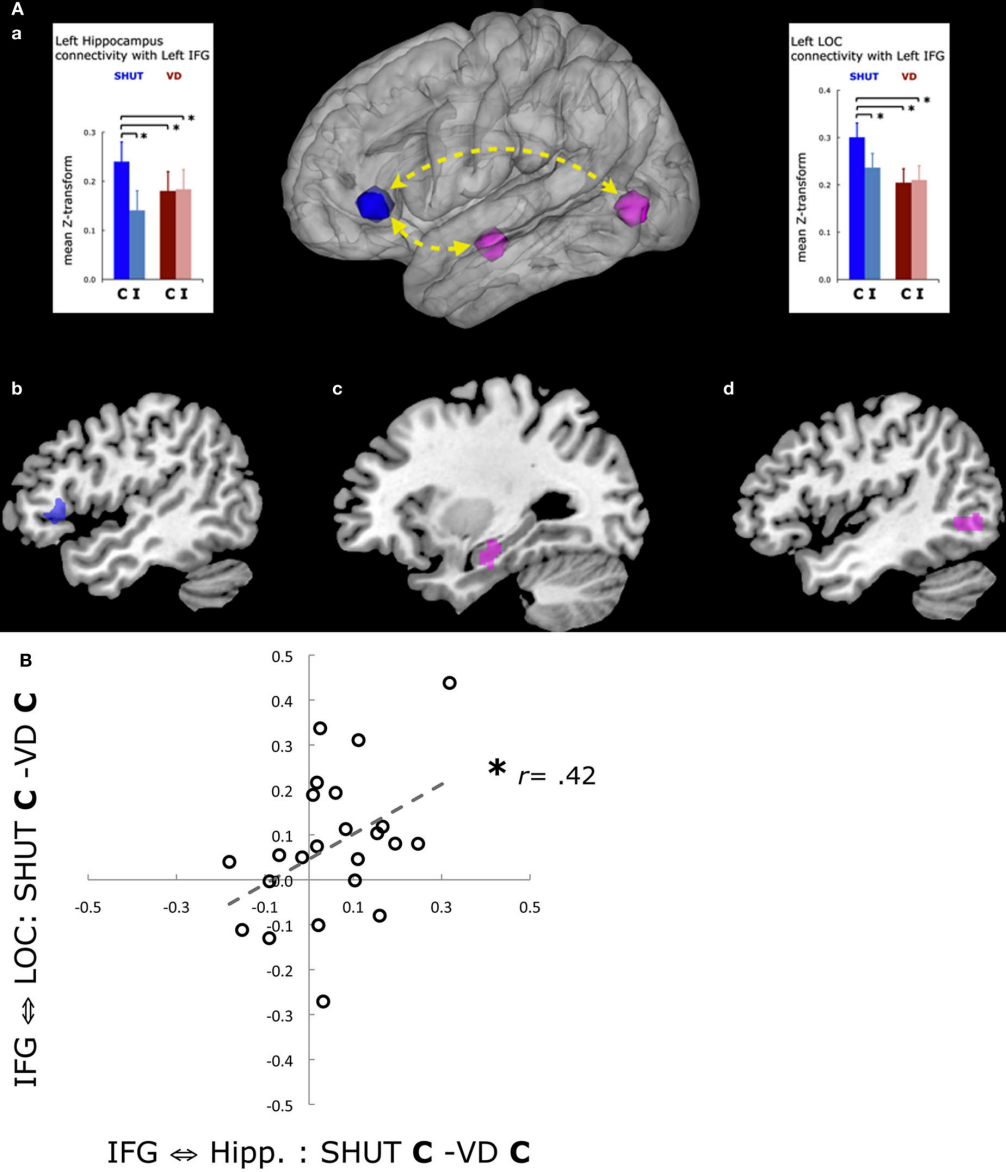
**(A)** fMRI results associated with visual distraction. The conjunction of functional connectivity with the left IFG was mapped by the whole-brain comparison of beta-series correlations seeded by the left hippocampal ROI identified in the univariate analysis and by the left LOC cluster identified in the independent functional localizer task (Wais et al., 2010). Further comparisons in this memory retrieval network revealed that functional connectivity was disrupted during VD correct, relative to SHUT correct. **(a)** A schematic of the network is shown with functional connectivity between the regions plotted as the mean z-score transformation of the beta-series correlations for each of four categories for responses to the targets. The network regions include: **(b)** the left IFG (blue); **(c)** the left hippocampus (violet); and **(d)** the left LOC (green). Error bars represent the standard error of the mean, and ^*^p < 0.05. **(B)** Disruption of functional connectivity in memory networks is associated with diminished episodic retrieval. A scatter plot shows the values for each participant in a regression analysis of functional connectivity between the left IFG and the left hippocampus ROIs (x-axis, SHUT correct vs. VD correct) and the left IFG and the left LOC ROIs (y-axis, SHUT correct vs. VD correct). The analysis revealed that reduced left IFG connectivity with the left LOC was correlated with reduced connectivity with the left hippocampus (Wais et al., 2010). Trend lines show the slope of significant correlations, and ^*^p < 0.05.

To assess the mechanism underlying the impact visual distraction has on episodic retrieval, we first interrogated the two hippocampal ROIs that were identified to subserve correct recall responses in the SHUT condition. This analysis revealed a differential impact by distraction in the VD condition such that the signal in the left hippocampus ROI was reduced in VD correct, relative to SHUT correct. The next step was a whole-brain, beta-series correlation analysis performed to assess functional networks including the hippocamal and LOC regions identified by the univariate analyses in the SHUT condition. Using network maps generated from these two seed regions, a contrast revealing greater functional connectivity in SHUT correct than SHUT incorrect trials identified a cortical network that included regions in the PFC, the insula and the posterior parietal cortex. A conjunction analysis of these network regions that supported episodic retrieval in SHUT revealed a single region in the left ventrolateral PFC [inferior frontal gyrus (IFG), BA45] that exhibited greater functional connectivity in common with the left hippocampus and the left LOC seed during SHUT correct than incorrect (Figure 3A). This left VLPFC region has been previously identified in studies utilizing univariate analysis as being a control region associated with selection of contextual information during LTM retrieval (Kahn et al., 2004; Dobbins and Wagner, 2005; Law et al., 2005; Wais et al., 2010; Wais, 2011). Moreover, the left VLPFC has also been identified in studies that mapped reinstatement of cortical encoding activity during later recognition tests (Wheeler and Buckner, 2004; Johnson et al., 2009).

Next, we evaluated the impact on this functional network from visual distraction during episodic retrieval. In comparisons of the left-lateralized hippocampus-VLPFC-LOC network between the SHUT and VD conditions, functional connectivity decreased in association with VD correct, relative to SHUT correct, and, critically, no longer supported episodic retrieval (i.e., functional connectivity was not different between VD correct and VD incorrect). Moreover, a regression analysis revealed that the change in network connectivity between SHUT correct and VD correct was correlated for an index of left VLPFC with left hippocampus connectivity and an index of left VLPFC with left LOC connectivity (Figure 3B). The results showed, therefore, that when VLPFC network connectivity decreased with the left LOC, it also decreased with the left hippocampus and that disruption of connectivity in this network was associated with diminished fidelity of episodic retrieval.

### Perturbation of left VLPFC

**Figure 4:**
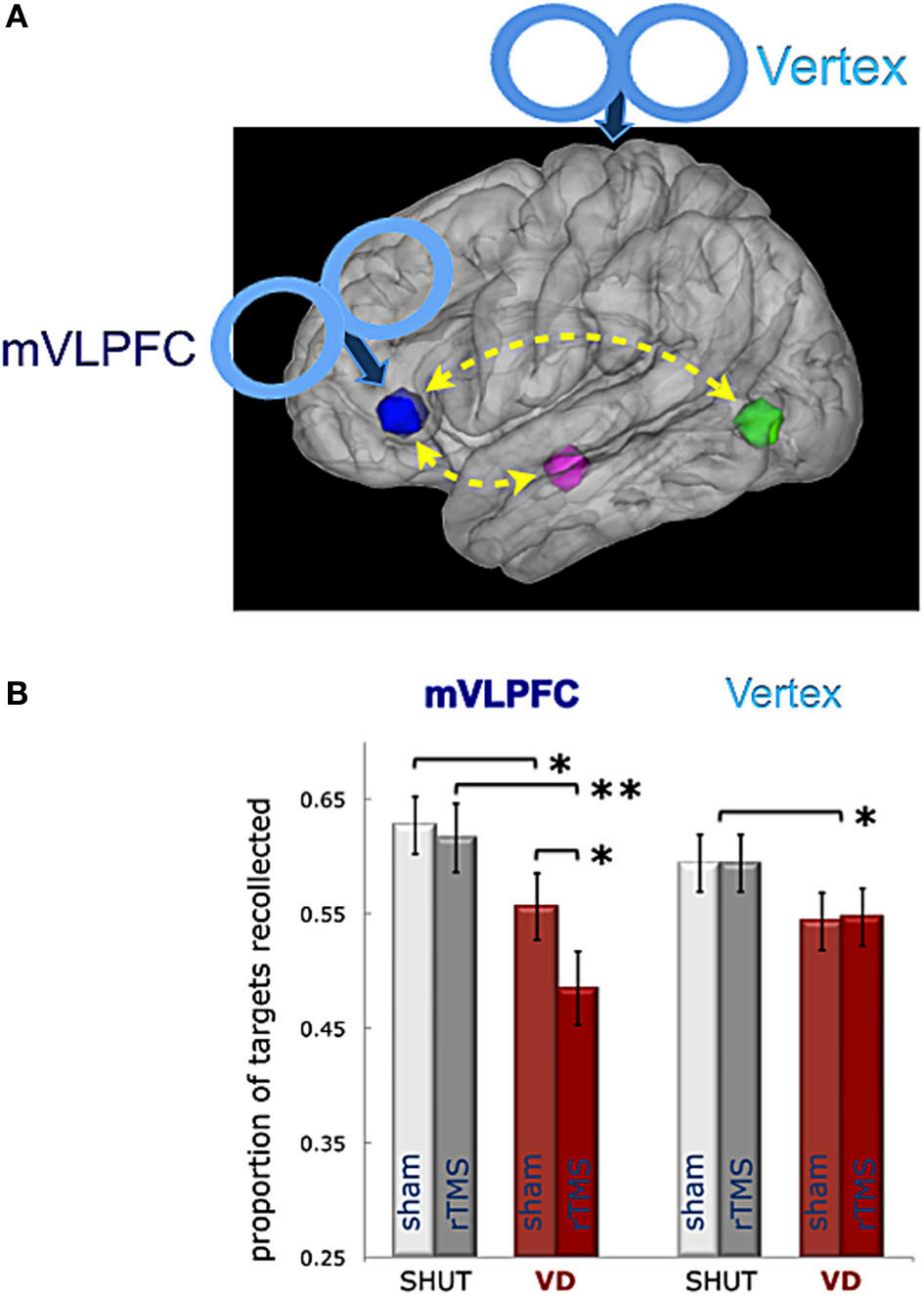
**Role of left VLPFC in episodic retrieval during visual distraction**. A schematic illustration **(A)** shows the rTMS targets located on a sagittal rendering of the MNI template brain, including the mVLPFC ROI (blue) functionally connected in a recollection network with the left hippocampus (magenta) and lateral occipital cortex (green), as represented in Wais et al. (2012a). **(B)** The mean proportion of targets given the correct count is shown in each experiment by condition (SHUT, VD) after sham or actual rTMS treatment. Results show an interaction of actual rTMS on episodic retrieval during visual distraction after VLPFC treatment, but not after Vertex treatment. Error bars represent the standard error of the mean; ^**^ indicates a difference between means *p* < 0.005; and ^*^ indicates a difference between means *p* < 0.05.

The mutual functional connectivity of the left VLPFC region with an object-selective region involved in visual imagery and a memory region critical for episodic retrieval suggests that the left VLPFC may serve as a source of cognitive control in a functional network necessary for the selection of contextual mnemonic details based on visual imagery. Based on the results from the fMRI study, the causal involvement of the left VLPFC ROI in episodic retrieval was assessed using repetitive transcranial magnetic stimulation (rTMS) to perturb normal function immediately prior to test blocks in the memory test (Wais et al., 2012a). Our approach incorporated two separate controls, such that the effects of actual rTMS perturbation could be compared to sham rTMS, (i.e., perturbation control when the rTMS pulse is not directed at the brain) and the effects of rTMS to the VLPFC could be compared to a cortical region not associated with LTM function or higher order cognition (i.e., vertex control). Thus, each participant engaged in two separate experiments—rTMS and sham rTMS applied at the left VLPFC and, on a different day, rTMS and sham rTMS applied at the vertex (Figure 4A).

The causal role of the VLPFC ROI in episodic retrieval was assessed in the SHUT and VD conditions by submitting the proportions of Correct cued-recall responses to a comparison between treatment and retrieval conditions. The results from a repeated-measures ANOVA of Site (VLPFC|vertex) × rTMS (sham|actual) × Condition (SHUT|VD) revealed a main effect of Condition and a significant interaction of Site × Condition. Correct responses decreased during VD, relative to SHUT, and this disruption of episodic retrieval was to a greater degree in the VLPFC experiment than the Vertex experiment (Figure 4B). The ANOVA also strongly suggested the interaction of rTMS × Condition, such that Correct responses were reduced during VD, relative to SHUT, to a greater degree after actual rTMS than sham. Moreover, the difference in Correct responses between conditions can be presented as an index of distractibility on episodic retrieval (i.e., SHUT Correct—VD Correct), and a comparison of this index between Sites revealed that the effect of distraction was exacerbated in the VLPFC experiment. Thus, the comparison of the distractibility index after actual rTMS, relative to that index after sham, further suggests that distraction was exacerbated by active rTMS to the left VLPFC.

### Summary of findings from neuroimaging

The fMRI study revealed for the first time that the fidelity of episodic retrieval declines in the presence of irrelevant external information and that this decline is associated with disrupted hippocampal function. Our interpretation of the fMRI results obtained during the SHUT condition is that the fidelity of episodic retrieval depends upon reinstatement of encoded representations for details relevant to memory goals, or visual imagery. This is consistent with results from previous fMRI studies that have shown reinstatement of activity associated with encoding visual stimuli when recognition was successful (Wheeler and Buckner, 2004; Johnson and Rugg, 2007; Johnson et al., 2009). However, the conclusions from prior research were limited to interpretations about subjective recollection and by the processing of visual memory cues concurrent with reinstatement of visual imagery processes engaged for the studied items. Our approach addressed these limitations by probing the recall of specific details of the memoranda when the participant's eyes were shut so that no external information was being processed during the memory retrieval process.

The study revealed the sensitivity of normal LTM operations to disruption by the presence of irrelevant environmental stimuli, such that the mere act of having eyes open to the surrounding environment decreases the accuracy of memory retrieval. Specifically, we found that a functional memory network involving the left hippocampus, PFC and LOC, which supports visual imagery and successful episodic retrieval when our eyes are closed, is disrupted by external distraction. This impact on performance and functional connectivity are likely mediated by capacity limitations in frontal control processes. In another study using rTMS to perturb function of the PFC node of the functional memory network, the results revealed that the left VLPFC has a direct role during retrieval of LTM in resolving competition between irrelevant external information and relevant mnemonic details. Limitations in processing capacity of prefrontal regions are a fundamental aspect in understanding the framework of cognitive control (Braver et al., 2009). The evidence in our studies revealed a critical role of the left VLPFC in the ability to reconstruct memories while interacting with our external environment.

### Distraction impairs categorization abilities in normal aging

The detrimental influence of distraction on LTM retrieval is now established, yet it is not as clear if irrelevant information impacts the underlying cognitive faculty for categorization learning. Categorization is the ability to discriminate key stimulus attributes according to abstract task rules (Ashby and Maddox, 2005), and this capability involves decision-making processes to sharpen the features of complex object representations (Freedman et al., 2003). Categorization, for example, underlies the ability to accept lemons, but reject tennis balls, as food. Categorization involves top-down control of visual attention to focus discrimination on the goal-relevant features of a stimulus during perception (Roy et al., 2010). In a new study, we examined the effects of distraction on categorization abilities in both younger and older adults, using an adaptive staircase approach to assess participants' discrimination of morphed prototype images in conditions with and without visual distractors (Wais and Gazzaley, in revision).

Psychology and neuroscience research suggest compatible models for mechanisms that integrate top-down and bottom-up processes to support sharpening of discrimination in categorization that underlies visual learning. For example, models for both a visuo-spatial sketchpad (Baddeley, 2010) and for neural activity ensembles as coherence fields (Serences and Yantis, 2006) propose that a junction in cognitive processing integrates goal-directed control of visual attention onto bottom-up representations of relevant perceptual information. This junction is thought to enable sharpening in object discrimination and may be a locus where the influence of visual distractors could interfere with top-down processes supporting visual learning. Precision of discrimination (i.e., sharpening goal-relevant representations) might be hindered when demands by top-down modulation networks that are engaged to suppress visual distraction overlap and interfere with the integration of top-down and bottom-up signals at the locus of sharpening of goal-relevant perceptual information.

Age-related effects of distractibility may also provide important insight about the processes and substrates underlying categorization abilities. A broad literature has proposed that WM decline in older adults is based on a combination of underlying factors, which include changes in basic capabilities for visual search (Hommel et al., 2004) and deficits in the ability to suppress irrelevant information (Hasher et al., 1999; Gazzaley et al., 2005a,b, 2008). Results from examinations of age-related changes for categorization capabilities are, however, equivocal (Filoteo and Maddox, 2004; Mayhew et al., 2010; Glass et al., 2012). If categorization performance is similar for both older and younger adults under well-controlled circumstances, but older adults' performance is disrupted by the presence of distractors, the finding would suggest that aging-related changes in the ability to discriminate goal-relevant perceptual features could be attributed in part to increased susceptibility to distraction.

**Figure 5:**
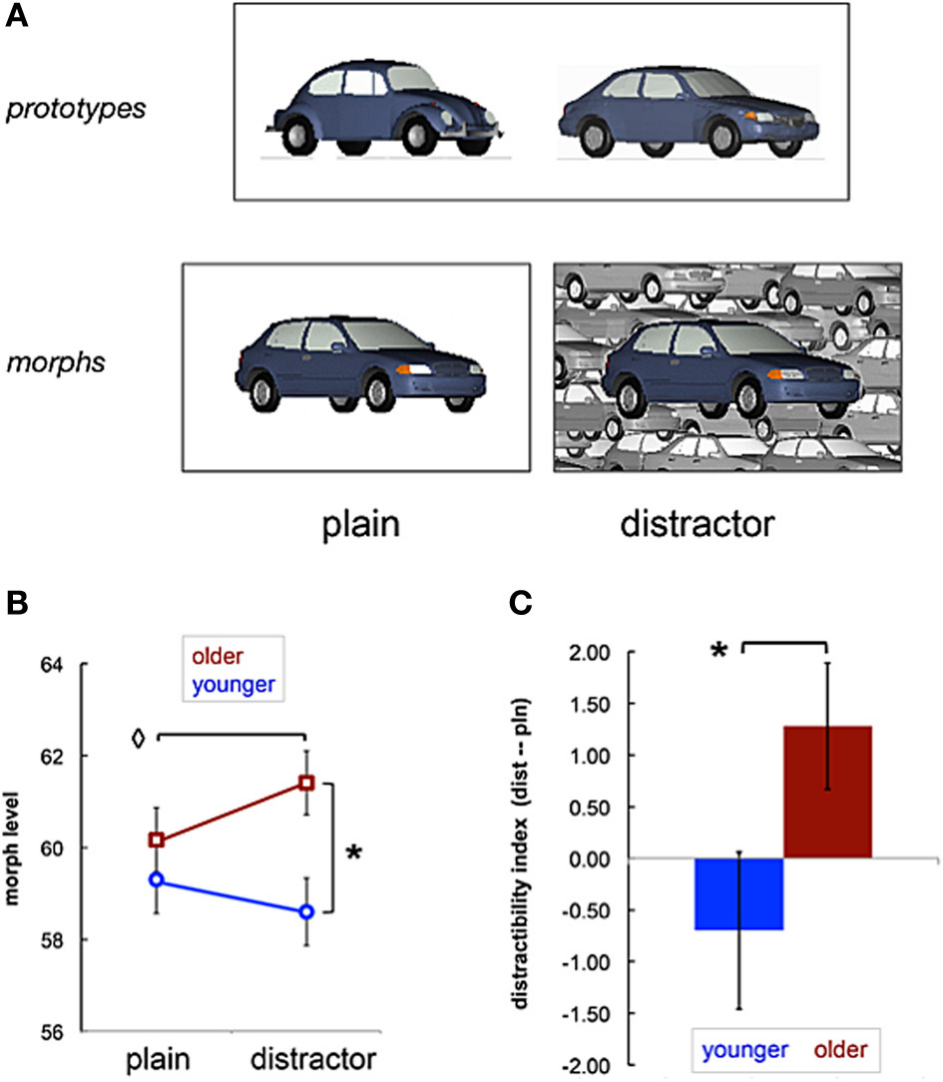
**Age-related influence of visual distraction on categorization learning**. The categorization procedure **(A)** presented a side-by-side pair of category prototypes, and then an exemplar morphed from the prototypes in blocks of either plain or distractor conditions. Results **(B)** for mean categorization thresholds for groups of older and younger adults showed a main effect of age and suggested that categorization learning declined for older adults in the distractor, relative to plain conditions. Comparisons between age groups for an index of distractibility **(C)** revealed that older adults were more susceptible to the negative impact of distraction during categorization than younger adults (*p* < 0.05). Error bars indicate the standard error of the mean; ^◊^ indicates a trend of difference between means, *p* < 0.06; and ^*^ indicates a difference between means, *p* < 0.05.

The categorization experiment used morphed visual prototype stimuli (Ashby and Maddox, 2011), with and without distraction, to assess participants' discrimination of relevant perceptual features. Participants were one group of 19 younger adults (9 males, age 20–29 years) and one group of 20 older adults (10 males, mean age = 68.2 ± 7.2 years), all of whom were tested for normal or corrected-to-normal vision in experiment orientation. On each trial, two different prototypes of an object category (i.e., two cars or two snowboards) were presented side-by-side and followed by presentation of a morphed exemplar, which the participant endorsed as belonging to one of the two prototype categories (Figure 5A). Each morphed exemplar was generated by integrating the feature information from 75 to 100 significant corresponding points on the category prototypes. The morph ratio (i.e., varying in difficulty from 75:25% up to 51:49%) changed according to an adaptive staircase algorithm with feedback that held accuracy constant at approximately 70%. Higher levels of morph ratio (i.e., 70% prototype A and 30% prototype B) were easier to categorize than lower levels of morph ratio (i.e., 48% prototype A and 52% prototype B). In the distractor condition, the morphed exemplars were centered on a grayscale collage composed from fragmented views of the respective category prototypes (i.e., irrelevant visual information). Participants' categorization threshold was assessed in terms of morph ratio, and their performance was compared between plain and distractor conditions.

Morph ratio was compared as a repeated measure of condition (plain|distractor) between groups (younger|older). The results showed a main effect of group, such that younger adults categorized at a lower morph ratio (i.e., better performance) than older adults. An interaction of age × condition revealed that older adults were more susceptible to visual distraction during categorization than younger adults. Critically, comparisons between age groups showed no difference in performance in the plain condition, but older adults categorized with a significantly higher morph ratio in the distractor condition than did younger adults. Results within the group of older adults suggested that distractor exemplars were categorized with a higher morph ratio than plain exemplars. We analyzed the basis for this pattern in the results by comparing the mean distractibility index between age groups. An index for each participant was calculated as morph ratio in the distractor condition minus morph ratio in the plain condition, such that a positive value showed a disruptive effect of distractibility (Figure 5B). An independent samples *t*-test (assuming unequal variances) showed that distractibility during categorization was greater for older than younger adults.

The study examined for the first time, to the best of our knowledge, the impact of distraction on categorization learning. We found that distractors did not affect categorization of morphed exemplars for younger adults. This finding reveals that top-down processes engaged to enhance representations of relevant stimulus features during categorization are undisturbed when additional control resources are required to suppress processing of irrelevant bottom-up information during the distractor condition (Lavie and de Fockert, 2005). Interestingly, older adults were just as able as younger adults to categorize morphed exemplars in the plain condition, a finding that is consistent with some other rule-based categorization learning results (Filoteo and Maddox, 2004; Mayhew et al., 2010; Glass et al., 2012). The interaction of age and distraction in the results, however, showed that concurrent demands to integrate information for categorization processing and to suppress bottom-up influences from irrelevant visual information disrupted performance for older adults, but did not affect performance for younger adults.

Visual categorization is a fundamental capability in higher cognition that involves sharpening the representations of relevant stimulus features in order to accept or reject the value of a stimulus for task goals (Ashby and Maddox, 2005). Sharpening the representation of relevant stimulus features depends on reciprocal processes that integrate bottom-up stimulus-driven information, mediated by primary visual regions, with top-down task-specific information, mediated by prefrontal decision-making regions (Freedman et al., 2003; Jiang et al., 2007). As integration of information from task goals and visual sensation proceeds with practice, learning improves the fidelity of relevant stimulus attributes so that finer and finer discriminations are successful. In this manner, selective visual attention guides improvement of the coherence of goal-relevant representations via-a-vis competing perceptual information (Serences and Yantis, 2006). Visual categorization with exemplars morphed from two prototypes is thought to be particularly demanding on the integration of top-down and bottom-up signals that successively tunes relevant stimulus features (Zeithamova et al., 2008).

Our interpretation of the results from the morphed prototype study is that categorization task demands instigated top-down control of visual attention in synchrony with updating and maintenance of WM processes (Freedman et al., 2003; Jiang et al., 2007), and older adults showed distractibility during these increased demands on top-down control that young adults did not. We propose that older adults' capability to focus visual attention on selective areas within complete object representations was diminished when concurrent demands to filter irrelevant visual information exceeded limited control resources. The locus of integration of top-down and bottom-up inputs that reciprocate through the putative hierarchy of visual perceptual processing to build an object representation has been characterized as a coherence field (Serences et al., 2005; Serences and Yantis, 2006). fMRI results show that regions of lateral parietal cortex mediate spatially selective sharpening within the coherence field associated with an object representation (Serences and Yantis, 2007).

Categorization under circumstances influenced by visual distraction involves increased processing of bottom-up visual information. The increased flow of bottom-up information may, in turn, increase demands on processes that mediate coherence fields and diminish the precision of relevant object representations. Although younger and older adults discriminated equivalent levels of morphed prototypes in our categorization condition without distraction, distractibility diminished older adults' discrimination performance. This novel finding, in particular, suggests that age-related distractibility during categorization may have more to do with interference on sharpening processes that involve the integration of visual attention and object representations than simply a deficit in top-down control of visual attention.

## Conclusions

Our findings about the effects of distractibility on the fidelity of memory retrieval raised important new questions about the control of attention to visual imagery that supports LTM. Heretofore, the disruptive influence of irrelevant environmental information was understood to diminish performance on task goals served by WM (Lavie and de Fockert, 2005). The results from novel studies reviewed here revealed that LTM retrieval is also susceptible to disruption from distraction. Our findings are distinct from the literature regarding affects on LTM from divided attention or dual-tasks (Troyer et al., 1999). Specifically, we found that mnemonic details represented via visual imagery were not as accessible in conditions when perceptual distractors were present as in controlled conditions. Yet, in all conditions, participants directed their full attention to memory retrieval goals. In other words, our findings show that bottom-up processing of irrelevant environmental information diminishes the accuracy of episodic retrieval, and separate studies found that this critical cost is domain general. Moreover, there is an ageing-related increase in the cost distractibility on episodic retrieval.

Evidence from neuroimaging elucidated the functional networks supporting episodic retrieval that are susceptible to disruption from the influences of environmental distraction. Although the key nodes for networks supporting the fidelity of LTM were identified in our studies (i.e., regions of the VLPFC, MTL, and LOC), the precise locus where information from bottom-up processes associated with distraction interferes with information represented from LTM stores is, as yet, unclear. A potential substrate where perceptual information might intersect with top-down selection and tuning processes necessary for representation of episodic details is illustrated by the notion of coherence fields (Serences and Yantis, 2006). Coherence fields are thought to be mediated by functionally networked regions at of the occipital, parietal and frontal cortices (Serences and Yantis, 2007).

We also recently examined the impact of visual distraction on categorization learning, using a task that is orthogonal to LTM retrieval yet very demanding on the fidelity of information represented in immediate memory (Jiang et al., 2007). The results showed that young adults' categorization performance was not affected by visual distraction, whereas older adults were susceptible to distraction during categorization. This ageing-related deficit in filtering out irrelevant distracting information during categorization is convergent with previous findings for visual WM (Gazzaley et al., 2005a,b; Clapp and Gazzaley, 2012). It may be the case that cognitive control resources, although limited, have the capability to resolve interference from distractors during tasks of moderate effort (i.e., calling on WM), but these resources are overwhelmed when additional processes associated with episodic retrieval are required. Indeed, remembering specific details has been shown a particularly effortful cognitive load (Atkinson and Juola, 1973). Age-related distractibility during categorization, therefore, may provide meaningful insight concerning the locus of interference of distractors on the fidelity of details represented from LTM.

### Conflict of interest statement

The authors declare that the research was conducted in the absence of any commercial or financial relationships that could be construed as a potential conflict of interest.
